# Metal‐Half‐Salen Chemistry for One‐Pot Mineralization on Hydrophobic Polymer Membranes

**DOI:** 10.1002/advs.76821

**Published:** 2026-08-02

**Authors:** Rou‐Ming Wen, Hao Ye, Ming‐Bang Wu, Lu‐Lin Ma, Qi‐Hui Ye, Zi‐Hao Yuan, Yi Xie, Juming Yao, Hao‐Cheng Yang

**Affiliations:** ^1^ School of Materials Science and Engineering Zhejiang Sci‐Tech University Hangzhou People's Republic of China; ^2^ Zhejiang Provincial Innovation Center of Advanced Textile Technology Shaoxing People's Republic of China; ^3^ College of Polymer Science and Engineering State Key Laboratory of Polymer Materials Engineering Sichuan University Chengdu People's Republic of China; ^4^ High‐Tech Organic Fibers Key Laboratory of Sichuan Province Chengdu People's Republic of China; ^5^ MOE Engineering Research Center of Membrane and Water Treatment Technology, and Key Lab of Adsorption and Separation Materials & Technologies of Zhejiang Province Department of Polymer Science and Engineering Zhejiang University Hangzhou People's Republic of China; ^6^ The “Belt and Road” Sino‐Portugal Joint Lab on Advanced Materials International Research Center for X Polymers Zhejiang University Hangzhou People's Republic of China

**Keywords:** metal ions, metal‐half‐Salen system, organic–inorganic composite membranes, salicylaldoxime, versatile one‐pot strategy

## Abstract

Organic–inorganic composite membranes combine the advantages of polymers and inorganic materials, yet their fabrication remains challenging due to poor interfacial compatibility, especially on chemically inert hydrophobic substrates. Here, we report a one‐pot strategy to construct robust organic–inorganic composite membranes via metal‐half‐Salen chemistry, which enables simultaneous coordination and hydrolysis under a unified mildly acidic environment. Salicylaldoxime is used to effectively chelate metal ions, including Fe^3+^, Zr^4+^, and Mn^2+^, forming stable complexes that adhere to hydrophobic membranes through hydrophobic interactions and initiate controlled inorganic mineralization. This thermodynamically regulated process resolves the pH mismatch typically encountered in conventional metal‐phenolic systems. The resulting FeOOH‐coated membranes exhibit multifunctional performance, including 98.3% uranyl ion removal via synergistic adsorption and catalysis, 99.8% dye degradation through photo‐Fenton reactions, and over 99.3% rejection of oil emulsions due to enhanced surface hydrophilicity. This work offers a universal route for fabricating high‐performance composite membranes, highlighting the potential of metal‐half‐Salen chemistry for material interfacial engineering.

## Graphical abstract

Here we report a versatile one‐pot synthetic strategy for organic–inorganic composite membranes, which leverages a metal half‐Salen system to precisely govern the competing kinetics of coordination and hydrolysis. In contrast to conventional two‐step metal‐phenol chemistries, our integrated approach substantially simplifies the fabrication process, enhances reaction efficiency, and minimizes chemical consumption and waste, offering a more sustainable and scalable manufacturing paradigm.

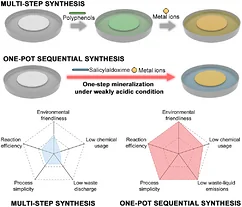

## 1 Introduction

Organic–inorganic composite membranes offer a unique combination of advantages by synergistically integrating the flexibility, processability, and cost‐effectiveness of organic polymers with the mechanical strength, thermal stability, and versatile functions of inorganic materials [1, 2, 3, 4, 5]. A critical aspect in fabricating these membranes is engineering a robust interface between the organic and inorganic components, which is particularly challenging for hydrophobic polymer membranes due to their inherent chemical inertness and weak interactions with inorganic materials [6, 7, 8]. Metal‐phenolic chemistry has emerged as a promising approach for enhancing polymer‐inorganic interactions in membrane fabrication [9, 10, 11]. Phenolic groups can efficiently coordinate with metal ions, forming a network on the polymer surface that not only strengthens the interaction but also acts as a nucleation site for inorganic material growth by, for example, hydrolysis [12, 13, 14]. In particular, for hydrophobic polymers, polyphenols interact with them via hydrophobic interactions, and the rapid formation of a cross‐linking network further reinforces interlayer adhesion [15, 16, 17]. However, conventional metal‐phenolic chemistry typically involves a two‐step process: phenolic groups coordinate with metal ions to create a conformal interlayer, followed by hydrolysis of the metal ions to form inorganic materials [18]. These steps typically require distinct pH conditions, complicating the fabrication process and limiting the practical applicability of this method [18, 19, 20, 21]. As shown in Scheme 1a, the chelation of polyphenols with metal ions typically requires an alkaline environment to prevent the protonation of phenolic hydroxyl groups [16, 22, 23], while the hydrolysis of metal ions generally occurs in a mildly acidic environment, enabling a relatively slow and controllable hydrolysis rate [24, 25, 26]. A significant challenge, therefore, lies in integrating the formation of the metal‐ligand cross‐linking interlayer with the hydrolysis of inorganic ions into a simple one‐pot process.

**SCHEME 1:**
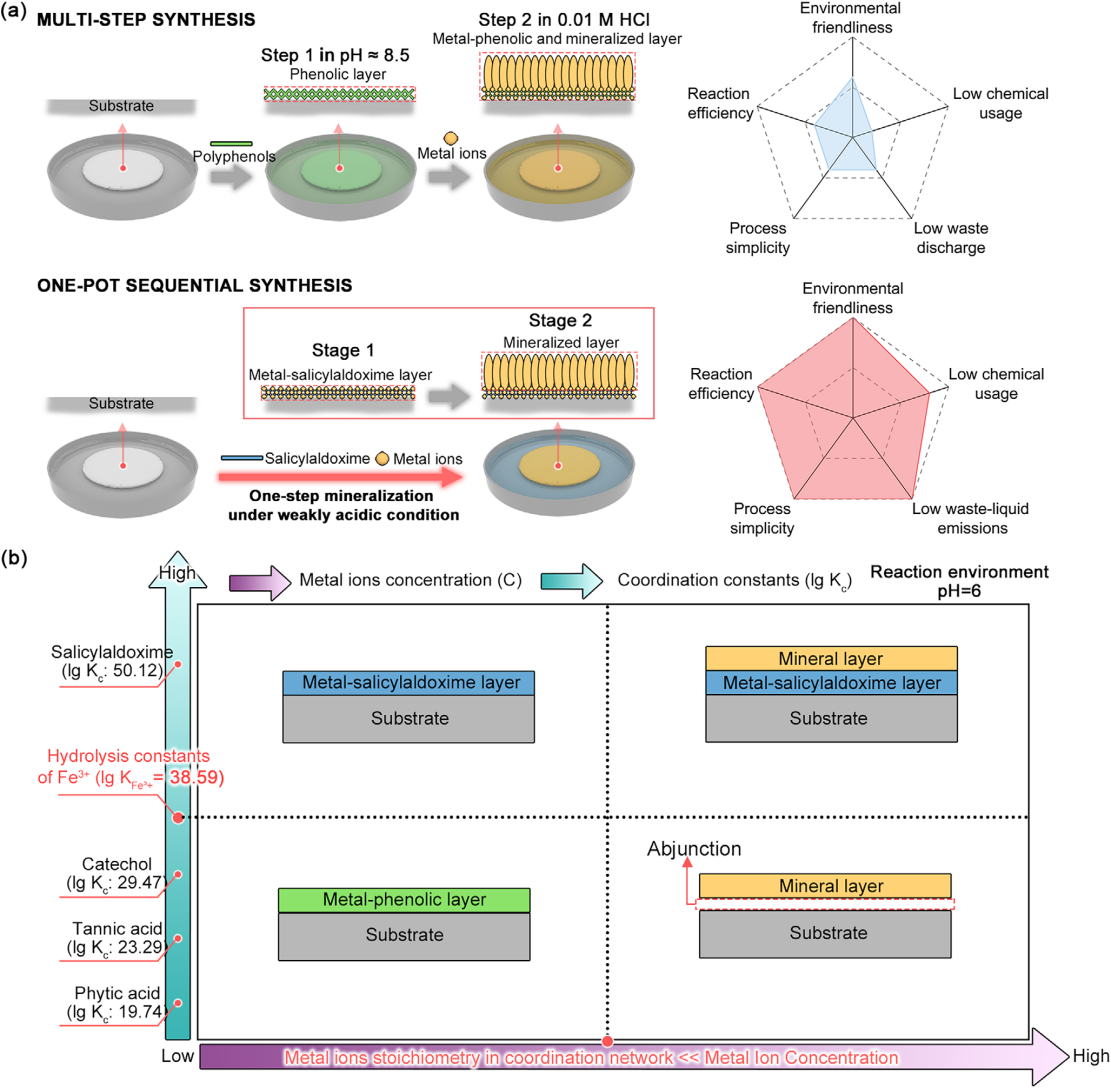
(a) Multi‐step and one‐pot sequential synthesis process flow and comparison. (b) Influence of phenol ligand coordination ability and metal ions concentration on mineral layer architecture.

Precise thermodynamic control over coordination and hydrolysis is crucial for achieving a simplified one‐pot synthesis of inorganic coatings on inert polymer surfaces. The reaction system should meet two essential conditions: (1) both reactions proceed within the same pH environment, and (2) the coordination complex should be more stable than the hydrolysis products [27, 28, 29]. Metal‐half‐Salen chemistry presents a promising approach to meet these requirements [30, 31, 32]. Compared with polyphenols, half‐Salen ligands provide both phenolate oxygen and imine nitrogen atoms for metal coordination, with the imine nitrogen being less pH‐sensitive and retaining strong coordinating ability under mildly acidic conditions [33, 34, 35, 36]. Mineralization in this pH regime also slows metal ion hydrolysis, which helps ensure coordination precedes hydrolysis and facilitates controlled coating deposition. Furthermore, the robust coordination between half‐Salen ligands and metal ions stabilizes the interfacial layer during mineralization [37, 38, 39].

Herein, we report a versatile one‐pot strategy for fabricating organic–inorganic composite membranes by precisely regulating the thermodynamic and kinetic evolutions of a metal‐half‐Salen system. From a thermodynamic perspective, the substantial disparity between the SA‐metal coordination constant and the metal ion hydrolysis constant serves as the driving force for the preferential formation of stable metal complexes. On the other hand, the mildly acidic environment (pH = 6) exerts kinetic control by slowing down the hydrolysis rate of metal ions, leading to coordination occurring prior to the mineralization. The aromatic moieties in Salicylaldoxime (SA) promote adhesion to the hydrophobic polymer surface via hydrophobic interactions, while the adsorbed SA‐metal complexes serve as nucleation sites for subsequent mineral growth [40, 41]. Compared with conventional multi‐step synthesis, the overall processing time is reduced by approximately 50%, and chemical reagent consumption and waste‐liquid emissions are reduced by nearly 75% and 90%, respectively (Table S1). The resulting FeOOH composite membranes exhibit excellent performance in uranium removal, dye degradation, and oil–water separation. Specifically, the membrane achieves a 98.3% removal efficiency for uranyl ions through a synergistic mechanism of adsorption and catalysis, a 99.8% degradation efficiency of dyes via photo‐Fenton reactions, and over 99.3% rejection of oil emulsions, attributed to its excellent hydrophilicity. This approach effectively resolves the inherent conflict between the optimal pH conditions for polyphenol‐metal chelation and controlled metal ion hydrolysis, offering a simplified route to advanced composite membrane fabrication.

## 2 Results and Discussion

Scheme 1b compares the reactions of SA and polyphenol ligands with metal ions. At low metal ion concentrations, both SA and other polyphenol ligands can successfully form coatings on the substrates. As the metal ion concentration increases, minerals grow and adhere robustly to the substrate in the case of SA, while the minerals detach from the substrate and precipitate within the bulk solution in polyphenol systems. Competition between coordination and hydrolysis determines the formation of cross‐linking layers, mineral coatings, or dispersed mineral particles in the solution. When the coordination constant exceeds the hydrolysis constant, a stable interlayer forms first, followed by the growth of minerals on it. Conversely, when hydrolysis dominates, metal‐ligand chelation lacks sufficient stability, leading to mineral precipitation in solution rather than deposition on the substrate. Specifically, the coordination constant between SA and Fe^3+^ significantly exceeds the hydrolysis constant of Fe^3+^ at pH = 6 (Figure 1a). In contrast, other polyphenols exhibit significantly lower coordination constants with Fe^3+^, leading to preferential hydrolysis and hydroxide precipitation before effective coordination. The strong affinity between SA and Fe^3+^ results in robust chelation, as shown in Figure 1b. Electrospray ionization mass spectrometry (ESI‐MS) results indicate that only coordination complexes are formed in the Fe^3+^‐SA system, consistent with previous reports in the literature (Figure S1) [42]. Notably, the imine nitrogen remains unaffected by protonation, enabling SA to chelate Fe^3+^ and form a stable complex that deposits onto the membrane surface under the weakly acidic conditions. Subsequent hydrolysis of the Fe^3+^ within this deposited complex initiates the growth of *β*‐FeOOH nanorods.

**FIGURE 1:**
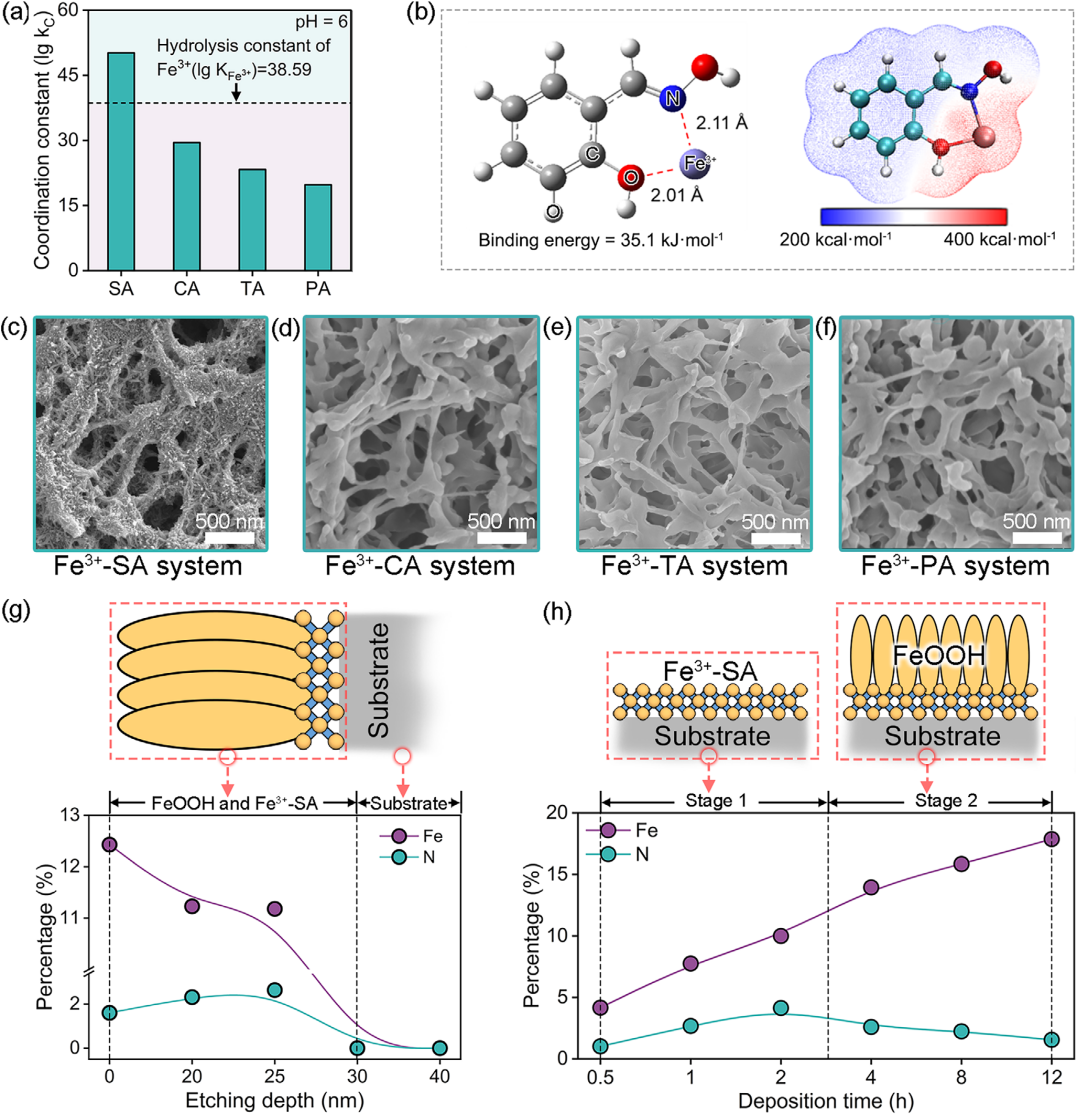
(a) Coordination constants of different phenolic ligands with Fe^3+^. (b) Molecular configuration and surface electrostatic potential distribution between Fe^3+^ and SA. (c–f) SEM images of PP membranes modified by different Fe^3+^/phenols systems. (g) Space‐resolved surface composition monitoring of the Fe^3+^‐SA system under different etching depths. (h) Time‐resolved surface composition monitoring of the Fe^3+^‐SA system under different deposition times. SA: Salicylaldoxime; CA: Catechol; TA: Tannic acid; PA: Phytic acid.

Figure 1c–f compares the surface morphologies of membranes prepared in different deposition systems. In the Fe^3+^‐SA system, distinct nanorods are evident on the surface of the polypropylene (PP) membrane, whereas no such nanorod structures are observed in the other Fe^3+^‐polyphenol systems, including Fe^3+^‐catechol (CA), Fe^3+^‐phytic acid (PA), and Fe^3+^‐tannic acid (TA). The hierarchical structure endows the membrane surface with promoted wettability (Figure S2). XRD pattern further indicates the formation of β‐FeOOH nanorods (Figure S3), and the characteristic peaks attributed to β‐FeOOH appear only in the Fe^3+^‐SA system. Thermogravimetric analysis (TGA) was conducted to quantify the mineral content on the membrane surface, indicating that the one‐pot strategy achieves a mineralization efficiency comparable to that of the conventional two‐step approach (Figure S4).

To further elucidate the formation process of the mineral coating, XPS analysis was conducted both along the coating depth and as a function of reaction time. Figure 1g shows the elemental distribution along the cross‐section of the surface mineral coating. N content increases toward the surface, while Fe content correspondingly decreases, indicating an Fe^3+^‐SA complex layer adjacent to the substrate, with a FeOOH mineral layer above it (Figure S5). Time‐resolved surface composition monitoring further demonstrates the formation of the Fe^3+^‐SA complex layer during the growth of *β*‐FeOOH (Figure 1h; Figure S6). Within the initial 2 h, both N and Fe contents increase with time, assigned to the formation of Fe^3+^‐SA complexes. Subsequently, the simultaneous decrease in N content and continuous increase in Fe content indicate that the growth of *β*‐FeOOH minerals dominates this stage. Mineral layer growth kinetics on substrates can be monitored via SEM (Figure S7). Both spectroscopic ellipsometry and TGA analysis confirmed the contribution of the mineralized layers to coating thickness and morphology (Figure S8).

The metal‐half‐Salen chemistry can also be applied to fabricate a variety of mineral coatings on diverse hydrophobic substrates. The coordination constants of SA with Fe^3+^, Zr^4+,^ and Mn^2+^ are all higher than the hydrolysis constants of these metal ions (Figure S9), satisfying the requirements of one‐pot mineralization mentioned above. The membrane color changes after the deposition of different minerals, including FeOOH, ZrO_2_, and MnO_2_ (Figure 2a). SEM images of the mineralized membranes show conformal, uniform coatings with nanoscale surface roughness (Figure 2b). XRD patterns further indicate the formation of β‐FeOOH, ZrO_2_, and MnO_2_ minerals on the membrane surfaces, respectively (Figure 2c). Both ATR‐FTIR and XPS spectra verify that the metal‐SA composite layer could efficiently mediate mineralization (Figures S10 and S11). The hyperhydrophilic transition of hydrophobic membranes after deposition also demonstrates the successful formation of an inorganic mineral layer (Figure S12).

**FIGURE 2:**
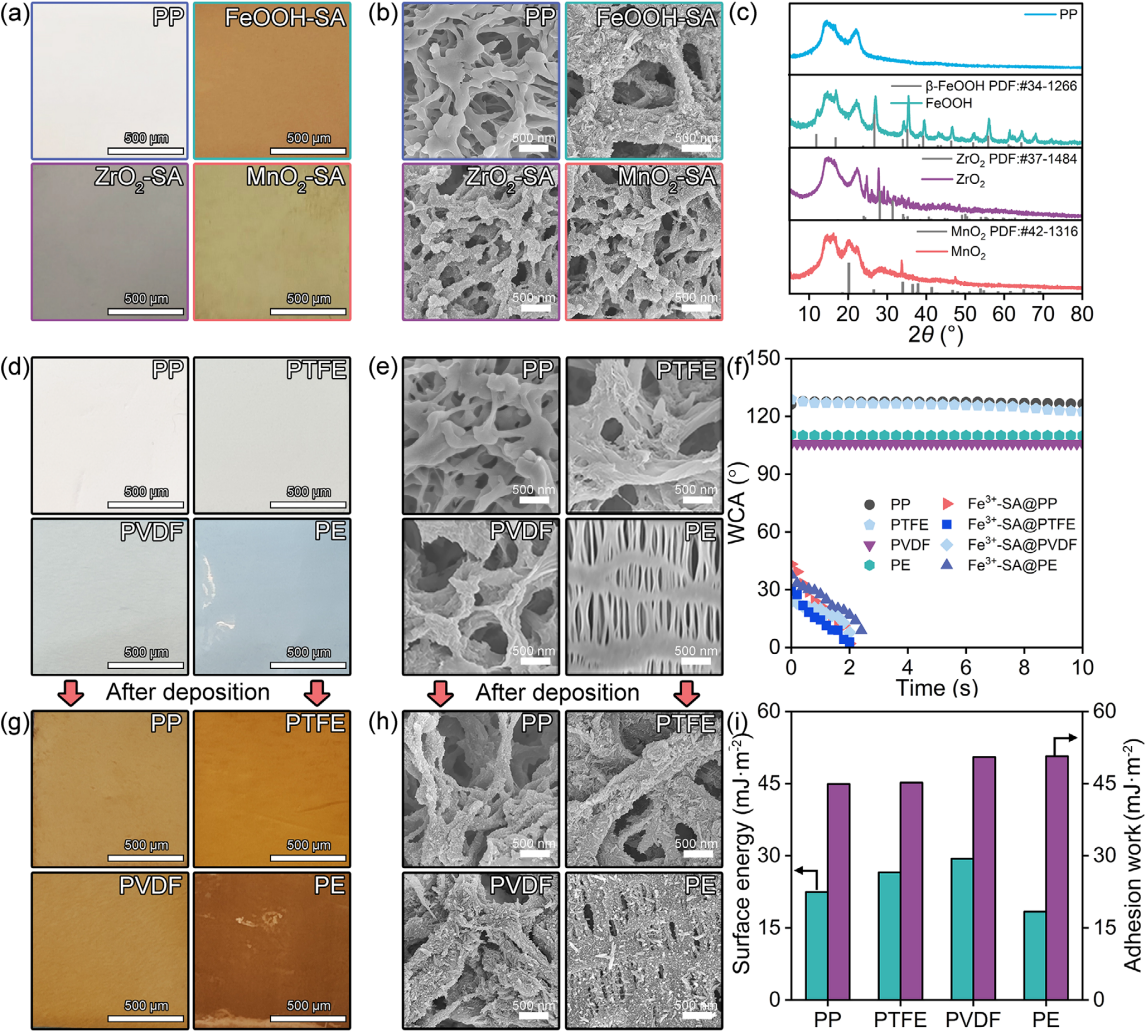
(a) Digital photograph, (b)SEM images and (c) XRD patterns of PP, Fe^3+^‐SA@PP, Zr^4+^‐SA@PP and Mn^2+^‐SA@PP. (d) Digital photograph and (e) SEM images of PP, PTFE, PVDF and PE before/after Fe^3+^‐SA system deposition. (f) Contact angles of PP, PTFE, PVDF and PE before/after Fe^3+^‐SA system deposition. (g) Surface energy and adhesion work of PP, PTFE, PVDF and PE.

The universality of this coating on diverse hydrophobic membrane substrates has also been demonstrated, as shown in Figure 2d–f. The hydrophobic interactions between metal‐half‐Salen complexes and hydrophobic surfaces contribute to the robust adhesion between mineral coatings and substrates, including PP, polytetrafluoroethylene (PTFE), polyvinylidene fluoride (PVDF) and polyethylene (PE) membranes. As shown in Figure 2d,e,g,h, *β‐*FeOOH nanorods grow effectively on these hydrophobic substrates, which are further conformed by ATR‐FTIR and XRD patterns (Figures S13 and S14). As mentioned previously, the FeOOH layer can endow hydrophobic membranes with excellent hydrophilicity, as revealed by the water contact angles (Figure 2f). The adhesion work of Fe^3+^‐SA layers on different low‐surface‐energy surfaces is calculated to demonstrate their robust adhesion (Figure 2i; Tables S2 and S3). The universality of this approach on other substrates is further confirmed by depositing the FeOOH layer onto a variety of flat, non‐porous surfaces, where a significant decrease in the water contact angle is observed (Figure S15). The mineral coating demonstrates excellent stability over a broad pH range and under saline, organic solvent, and oxidative environments, with negligible metal ion leaching (Figure S16).

The *β*‐FeOOH nano‐coating confers multiple functions to the membrane, including photocatalytic activity, antibacterial property, and oleophobicity (Figure 3a). These features enable the mineralized membranes to serve in diverse applications such as uranium extraction, organic pollutant degradation, sterilization, and oil–water separation.

**FIGURE 3:**
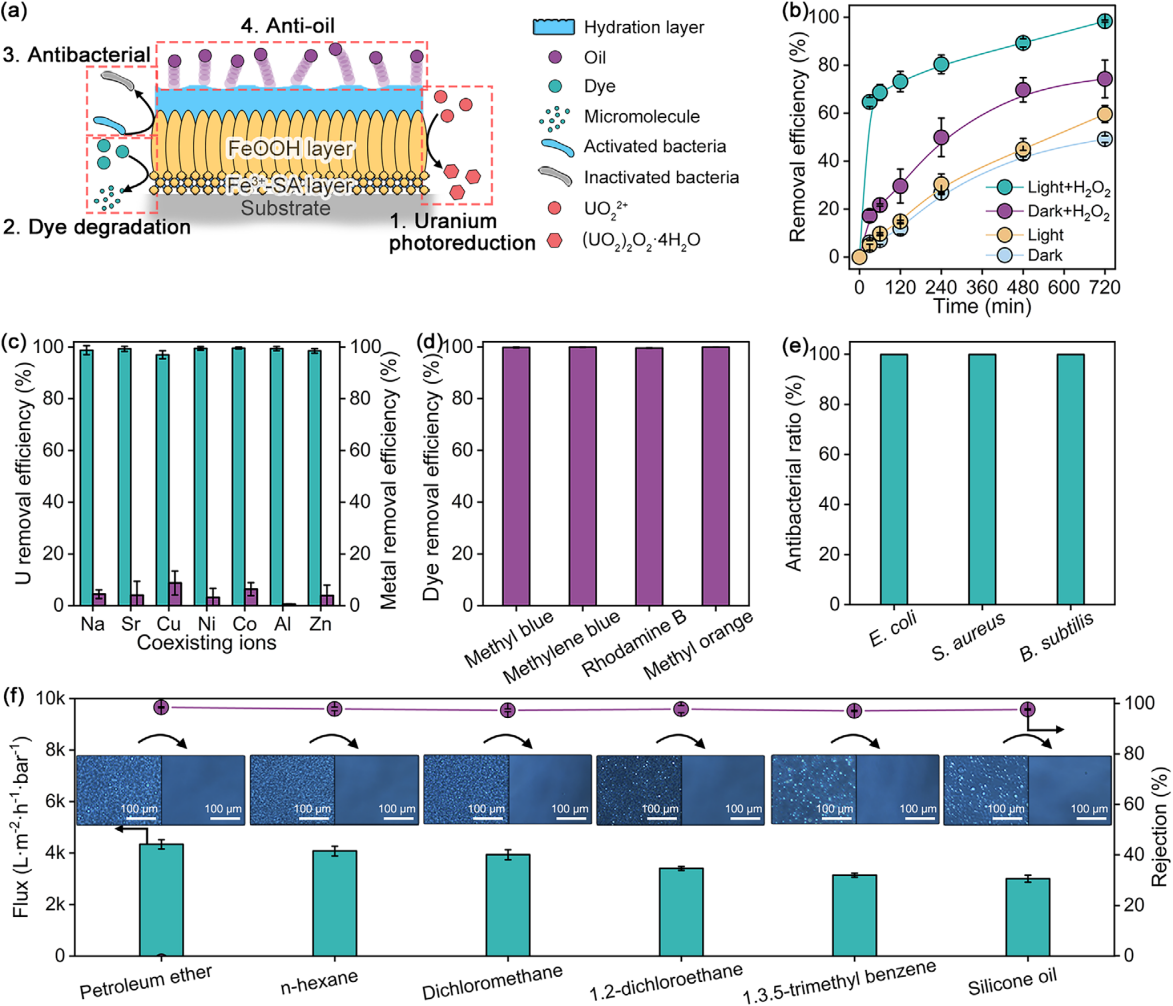
(a) Multifunctional water membrane treatment platform based on the Fe^3+^‐SA system. (b) Dynamic uranium extraction processes in different atmospheres (U(VI) concentration: 50 mg·L^−1^, volume: 50 mL, H_2_O_2_: 9.8 mM, *β*‐FeOOH‐coated membrane mass: 10 mg). (c) Removal efficiency of *β*‐FeOOH‐coated membrane for various metal ions (U(VI) concentration: 50 mg·L^−1^, metal ion concentration: 50 mg·L^−1^, volume: 50 mL, H_2_O_2_: 9.8 mM, *β*‐FeOOH‐coated membrane mass: 10 mg). (d) Dye degradation performances of *β*‐FeOOH‐coated membrane (dye concentration: 20 mg·L^−1^, volume: 50 mL, H_2_O_2_: 9.8 mM, *β*‐FeOOH‐coated membrane mass: 10 mg). (e) Antibacterial performance of *β*‐FeOOH‐coated membrane. (f) Oil–water separation performances of *β*‐FeOOH‐coated membrane (V_oil_: 1%, SDS: 20 mg·L^−1^). Insets are optical microscope photographs of the Oil–water solution before/after filtration.

Uranium mining generates substantial volumes of uranium‐contaminated wastewater, posing serious ecological and public health risks [43, 44]. The *β*‐FeOOH mineral layer provides abundant active sites, allowing the composite membrane to adsorb uranyl ions from solution effectively (Figure S17). The chemisorption‐dominated β‐FeOOH‐loaded membrane exhibits a theoretical saturation adsorption capacity of up to 260.3 mg·g^−1^. (Figure S18, Tables S4 and S5). In addition, *β*‐FeOOH is a narrow‐bandgap semiconductor catalyst with broad light absorption that can promote uranyl removal through photocatalytic reactions (Figure S19). The *β*‐FeOOH‐coated membrane exhibited excellent uranium removal performance in the self‐made photocatalytic system (Figure S20). Under the initiation of H_2_O_2_, the *β*‐FeOOH‐coated membrane achieved 65.8% removal efficiency within 30 min and 98.3% after 720 min (Figure 3b; Figure S21). Importantly, this process effectively converts soluble uranyl ions (UO_2_^2+^) into a highly stable and water‐insoluble crystalline phase, specifically (UO_2_)O_2_·4H_2_O (Figure S22). The membrane maintained stable uranium‐removal performance across various complex water environments (Figure S23). Notably, after six catalysis‐desorption cycles, the uranium removal and desorption efficiency remained above 83.2% and 88.2%, respectively (Figure S24). The *β*‐FeOOH‐coated membrane has exceptional selectivity for uranyl ions even in multi‐ion interference environments (Figure 3c).

*β*‐FeOOH is well known for its excellent photo‐Fenton catalytic activity, which enables the generation of abundant radicals for the degradation of organic pollutants [45] (Figure S25). Under simulated solar irradiation, the introduction of H_2_O_2_ significantly amplified the degradation activity of the mineralized membrane against various dye pollutants, yielding removal efficiencies exceeding 98.9% (Figure 3d; Figure S26). After treatment, the characteristic peaks of the dyes nearly disappeared in the UV–vis spectra (Figure S27), and the dye solutions turned colorless.

Bacterial contamination poses a severe threat to water safety [46]. SA acts as a potent broad‐spectrum antimicrobial agent, effective against both Gram‐negative and Gram‐positive strains [47]. Mechanistically, the abundant phenolic hydroxyl groups in SA interact with the bacterial cell envelope, compromising the permeability of the lipid bilayer. This disruption induces the irreversible leakage of intracellular electrolytes and macromolecules, ultimately triggering bacterial cell death. Specifically, the *β*‐FeOOH‐coated membrane demonstrated strong inhibitory effects against *Escherichia coli*, *Staphylococcus aureus*, and *Bacillus subtilis* compared with pristine *β*‐FeOOH and basement membrane, achieving bacterial suppression rates exceeding 99.8% (Figure 3e; Figures S28 and S29). Even when treating uranium‐containing solutions with varying bacterial concentrations, the mineralized membrane maintains uranium removal efficiencies over 90% (Figure S23c).

Beyond the catalytic functions, the hierarchical structure and hydrophilicity of the mineral coating also endow the membrane surface with good anti‐oil properties, which can find applications in oil–water separation. In this work, the mineralized membranes perform above 140° underwater oil contact angles for a variety of oils (Figure S30). In a pure water system, the water flux of the *β*‐FeOOH‐coated membrane is above 4834.2 L·m^−2^·h^−1^·bar^−1^ (Figure S31). During the dead‐end filtration process, the *β*‐FeOOH‐coated membrane achieved a separation efficiency exceeding 99.3%, with a maximum water flux reaching 4338.39 L·m^−2^·h^−1^·bar^−1^ (Figure 3f). Remarkably, the membrane achieves self‐cleaning through a facile photo‐Fenton reaction, utilizing the generated reactive oxygen species to degrade internal oil foulants into small molecules and fully restore its flux (Figure S32). Even in the presence of strongly adhesive contaminants such as proteins, the *β*‐FeOOH‐coated membrane preserved stable permeability and antifouling performance (Figure S33).

## 3 Conclusion

In summary, we present a universal one‐pot strategy for synthesizing organic–inorganic composite membranes that integrates coordination and mineralization processes within a single step. This approach utilizes a metal–half‐Salen system to simultaneously regulate coordination network formation and hydrolytic mineral growth, overcoming the incompatibility between conventional coordination and mineralization conditions. The strong chelation ability of the SA ligand enables broad adaptability of this approach to various mineral systems and hydrophobic polymeric substrates. The resulting mineral layer not only transforms the membrane surface from hydrophobic to superhydrophilic but also imparts multiple functional attributes, including enhanced mechanical stability, self‐cleaning antifouling capability, and photocatalytic activity. These features allow the membranes to achieve near‐complete removal of uranyl ions, organic dyes, and oil–water mixtures. Furthermore, the robust interfacial coupling between the Fe^3+^–SA coordination network and the *β*‐FeOOH mineral phase ensure long‐term structural integrity and stable performance under complex aqueous conditions and repeated operation cycles. This one‐pot mineralization strategy provides a versatile platform for fabricating high‐performance organic–inorganic membranes and offers new insights into the synergistic integration of separation and photocatalytic functions in advanced multifunctional membrane systems.

## 4 Methods

### 4.1 Materials

Salicylaldoxime (C_7_H_7_NO_2_, 98%), Catechol (C_6_H_6_O_2_, 99.5%), Phytic acid (C_6_H_18_O_24_P_6_, 90%), Tannic acid (C_76_H_52_O_46_, 98%), and Iron (III) chloride hexahydrateand (FeCl_3_·6H_2_O, 99%) were purchased from Shanghai Macklin Biochemical Technology Co., LTD. Polypropylene membranes (PP) with average pore sizes of 0.25 µm were provided by Membrana GmbH (Germany). Polyvinylidene fluoride (PVDF, 0.22 µm), polytetrafluoroethylene (PTFE, 0.22 µm), and polyethylene (PE, 0.22 µm) microfiltration membranes are commercial products of Haining Delv New Materials Technology Co., Ltd. (China). Manganese sulfate monohydrateand (MnSO_4_·H_2_O, 99%), zirconium sulfate (ZrSO_4_, 98%), acetic acid (HAc, 99%), tris (hydroxymethyl) aminomethane (Tris, 99%), and sodium acetate anhydrous (NaAc, 99%) were obtained from Shanghai Aladdin Biochemical Technology Co., LTD (China). Uranium hexahydrate nitrate [UO_2_(NO_3_)_2_·6H_2_O] was bought from Beijing HWRK Chem Co., LTD (China).

### 4.2 Characterization

Microscopic morphologies were obtained by scanning electron microscopy (SEM, Hitachi S4800, Japan). X‐ray diffraction (XRD, Panalytical X'Pert'3 Powder), Attenuated total reflectance Fourier transform infrared spectroscopy (ATR‐FTIR, Nicolet IS50), and X‐ray photoelectron spectroscopy (XPS, Thermo Scientific K‐Alpha) were carried out to obtain the chemical structures. The coating thickness was determined by an ellipsometer (Accurion GnbH, Germany). The water contact angles were measured by a contact angle meter (Dataphysics OCA20). The concentrations of uranium and other metal ions were recorded by an inductively coupled plasma‐optical emission spectrometer (ICP‐OES, PerkinElmer 8300). UV–vis absorption of the solutions was measured with an UV spectrophotometer (HITACHI UH4150, Japan). The contents of various oils were detected by the total organic carbon analyzer (TOC, Veolia, GE Sievers InnovOx ES, France). Diffuse reflectance UV–vis spectroscopy (HITACHI UH4150, Japan) was conducted to detect the optical response. Electron spin resonance spectroscopy (ESR, Bruker A300‐12, Germany) was used to determine the contents of free radicals in solution.

### 4.3 Fabrication of Functional Coatings

The hydrophilic modification of hydrophobic membranes was achieved by a one‐pot sequential synthesis method. First, the hydrophobic membranes were pre‐wetted in ethanol to remove impurities. Meanwhile, NaAc/HAc (100 mM) and Tris/HCl (100 mM) buffers with different pH values were prepared. Then, salicylaldoxime (SA) and the metal salt were dissolved in the above buffer solution with different mass ratios. The pre‐treated membranes were then immersed in the mixed solution and placed in a shaker (180 rpm, 25°C) for a designed time. After the reaction, the membrane was removed, rinsed with ultrapure water, and vacuum dried at 30°C for 3 h before use.

### 4.4 Calculation of Stability Constants

The stability constants of metal‐phenol coordination complexes were determined using the molar ratio method [48, 49]. Initially, metal ion solutions were prepared in various pH buffer environments. The reaction produced a series of ligand compounds with varying ratios by varying the concentration of the SA solutions. Subsequently, UV–vis spectrophotometric measurements of solution absorbance enabled the determination of the stoichiometric ratio between metal ion and phenolic ligands via the slope ratio method, thereby deriving the stability constant of the coordination compound.

The coordination reaction between Fe^3+^ and phenol can be succinctly expressed as:

(1) $$R+\mathrm{nM}\;⇌RM_{n}$$

The stability constants:

(2) $$K_{C}=\frac{\left[MR_{n}\right]}{\left[M\right]{\left[R\right]}^{n}}$$

where *K_C_* is the coordination constant of the phenol ligand to the metal ion; R is the phenol ligand, and M is the metal ion.

The hydrolysis reaction can be expressed as:

(3) $$M^{n+}+{\mathrm{nOH}}^{-}\to M{\left(\mathrm{OH}\right)}_{n}↓$$

The hydrolysis constants (K_M_):

(4) $$K_{M}=\frac{1}{\left[M^{n+}\right]{[OH^{-}]}^{n}}$$

where M is the metal and n is the charge number.

### 4.5 Molecular Electrostatic Potential Analysis

Quantum‐chemical calculations of the molecule using Gaussian16 yield optimized molecular configurations and electron‐cloud distributions. The electrostatic potential distribution of the molecule can then be mapped from this using Multiwfn in combination with VMD.

### 4.6 Calculation of Adhesion Work for Membranes

The adhesion work of membranes studied here was evaluated by the Owens‐Wendt method [4, 50, 51, 52], based on the experimentally determined intrinsic contact angles of water and diiodomethane (CH_2_I_2_).

(5) $$\sqrt{\gamma_{water}^{d}}\sqrt{\gamma_{sv}^{d}}+\sqrt{\gamma_{water}^{p}}\;\sqrt{\gamma_{sv}^{p}}=\frac{\left(1+cos\left(\theta_{water}\right)\right)\gamma_{water}}{2}\;$$

(6) $$\sqrt{\gamma_{diiodomethane}^{d}}\;\sqrt{\gamma_{sv}^{d}}=\frac{\left(1+cos\left(\theta_{diiodomethane}\right)\right)\gamma_{diiodomethane}}{2}\;$$

where the dispersive (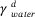
= 21.8 mN·m^−1^) and polar components (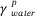
= = 51.0 mN·m^−1^) of water, and those of diiodomethane (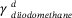
= 45.8 mN·m^−1^ and 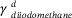 = 48.5 mN·m^−1^), were taken from literature.

### 4.7 Calculation of Adhesion Work for Coatings

The adhesion work of the coating was calculated by the contact angle method using the following equations [53, 54].

(7) $$\begin{array}{ccc} W_{wos} & = & 2\left(\gamma_{w}^{d}+y_{w}^{p}+\sqrt{\gamma_{s}^{d}\gamma_{f}^{d}}+\sqrt{\gamma_{s}^{p}\gamma_{f}^{p}}-\sqrt{\gamma_{s}^{d}\gamma_{w}^{d}}\right.\\ & & \left.-\sqrt{\gamma_{s}^{p}\gamma_{w}^{p}}-\sqrt{\gamma_{f}^{d}\gamma_{w}^{d}}-\sqrt{\gamma_{f}^{p}\gamma_{w}^{p}}\right) \end{array}$$

where $\gamma_{w}^{d}$ $\gamma_{s}^{d}$, $\gamma_{f}^{d}$ are the nonpolar components of surface tension for water, membrane, and coating, and $\gamma_{w}^{p}$, $\gamma_{s}^{p}$, $\gamma_{f}^{p}$ are the polar components correspondingly.

We determined the contact angle between water and diiodomethane (CH_2_I_2_) on the material to obtain the $\gamma_{f}^{d}\;$ and $\gamma_{f}^{p}$. The polar and nonpolar components of the surface tension of the coating can be calculated as follows:

(8) $$\cos\theta =-1+2\left(\frac{\sqrt{\gamma_{s}^{p}\gamma_{l}^{p}}+\sqrt{\gamma_{s}^{d}\gamma_{l}^{d}}}{\gamma_{l}}\right)$$

The measured contact angles are θ (H_2_O) = 64.4° and θ (CH_2_I_2_) = 37.5°. Finally, we obtained $\gamma_{f}^{d}$= 33.6 mJ·m^−2^, $\gamma_{f}^{p}$= 12.4 mJ·m^−2^, and Equation (7) can be converted to Equation (9):

(9) $$W_{wos}=2.68\sqrt{\gamma_{s}^{d}}\;-\;7.40\sqrt{\gamma_{s}^{p}}+41.61$$

### 4.8 Batch Adsorption

The modified membrane was added to the uranyl solution and placed on a shaker at 25°C in the dark for adsorption experiments. The uranyl concentration was measured by ICP‐OES. The adsorption capacity (q_t_) was calculated using the following equation:

(10) $$q_{t}=\frac{V(C_{0}\;-\;C_{e})}{m}\;$$

where C_0_ (mg L^−1^) and C_t_ (mg L^−1^) are the uranium concentrations at initial and designed time, respectively; V (L) is the volume of solution and m (g) is the quality of the membrane.

The pseudo‐second‐order Equation (9) and pseudo‐first‐order Equation (10) kinetic models were used to confirm the adsorption activities:

(11) $$\frac{t}{q_{t}}=\frac{1}{k_{2}q_{e}^{2}}+\frac{t}{q_{e}}$$

(12) $$ln\left(q_{e}\;-\;q_{t}\right)=lnq_{e}\;-\;k_{1}t$$

where q_e_ (mg g^−1^) is the capacity at equilibrium time; k_1_ (g mg^−1^ min^−1^) and k_2_ (min^−1^) are the rate constants for the pseudo‐second‐order and pseudo‐first‐order models, respectively.

The Langmuir Equation (11) and Freundlich Equation (12) models were applied to fit the equilibrium isotherm by the following equation:

(13) $$\frac{C_{e}}{q_{e}}=\frac{C_{e}}{q_{m}}+\frac{1}{k_{3}q_{m}}$$

(14) $$\mathrm{lg}q_{e}=\mathrm{lg}k_{4}+\frac{1}{n}\mathrm{lg}C_{e}$$

where C_e_ (mg·L^−1^) is the uranium concentration at equilibrium time; q_m_ is the saturated adsorption capacity (mg·g^−1^); k_3_ (L·mg^−1^) is the equilibrium constant; k_4_ is the indicator, which is close to adsorption capacity; 1/n is a function of the strength of adsorption in the adsorption process.

### 4.9 Photocatalyst

Subsequently, 9.8 mM H_2_O_2_ and a *β*‐FeOOH‐coated membrane were added to the configured solution and placed in a photochemical reaction apparatus (XPS‐7, Nanjing) for the uranium extraction experiment. The uranium extraction performance of modified membranes was systematically investigated by adjusting different environmental conditions. The extraction rate was calculated using the following equation:

(15) $$\mathrm{Extraction}\;\mathrm{rate}\;=\frac{C_{0}-C_{t}}{C_{0}}\times \;100\%$$

where C_0_ (mg·L^−1^) and C_t_ (mg·L^−1^) are uranium concentrations at initial and catalytic time.

### 4.10 Ion Selectivity

Equal concentrations of conventional cations‐spiked solution (UO_2_^2+^, Na^+^, Sr^2+^, Cu^2+^, Ni^2+^, Co^2+^, Al^3+^, Zn^2+^) were prepared and then added to the *β*‐FeOOH‐coated membrane for extraction under light conditions to study selectivity. Ion concentrations were measured by ICP‐OES after reaction equilibrium. The metal ions removal ratio (%) was presented as follows:

(16) $$R_{metal\;ions}=C/C_{0}$$

where R_metal ions_ are the removal ratios of metal ions; C_0_ and C are the uranium concentration before and after catalysis, respectively, mg·L^−1^.

### 4.11 Cycling Performance

After catalysis, the modified membrane was collected and added to a NaHCO_3_ solution to elute the attached uranium. Subsequently, the renewed photocatalysts were washed with pure water, dried under vacuum, and used in the next cycle. The uranyl concentration of the eluate was measured by ICP to determine the membrane's uranyl extraction rate.

### 4.12 Dynamic Extraction Experiments

Dynamic uranium extraction tests were carried out using a self‐made photocatalytic system (Figure S20) under visible‐light illumination (λ > 420nm, 500W Xe light source). Initially, the *β*‐FeOOH‐coated membrane was immobilized on the photocatalytic device, and 200 mL of uranium solution (pH = 6) at 50 ppm was prepared, followed by the addition of H_2_O_2_ (9.8 mM) to the solution. The uranium extraction performance of the *β*‐FeOOH‐coated membrane was evaluated by performing photocatalytic experiments in different conditions using a dynamic water circulation system.

### 4.13 Evaluation of Antibacterial Property

Before the performance assay, the cultured bacteria (*S. aureus*, *E. coli*, and *B.subtilils*) were redissolved in a 0.85 wt.% NaCl solution at a concentration of 10^5^ CFU mL^−1^. Subsequently, 5 mg of *β*‐FeOOH‐coated membrane was added to separate solutions containing different bacteria. After 24 h of incubation in a bacterial incubator, 100 µL of the bacterial solution (10^5^ CFU mL^−1^) was extracted and evenly spread on solid medium, which was then incubated at 37°C with 70% humidity. Colony growth on the medium surface was observed 12 h later to assess the modified membrane's bacterial inhibition efficiency. The bactericidal rate is calculated using the following equation:

(17) $$\mathrm{Bactericidal}\;\mathrm{rate}\;=\frac{C\;-C_{t}}{C_{0}}\times \;100\%\;$$

where C is the bacterial concentration after treatment of the *β*‐FeOOH‐coated membrane, and C_0_ is the initial bacterial concentration.

To further investigate the antimicrobial properties of the *β*‐FeOOH‐coated membrane, it was subjected to a uranium extraction test in bacterial solutions. A separate 50 mL of uranyl ion solution (50 mL L^−1^, pH 6) containing gradient concentrations of *E. coli* and *S. aureus* was prepared. The modified membrane was then introduced to conduct photocatalytic uranium extraction experiments. After reaching equilibrium, the concentration of uranium in the solution was measured using ICP‐OES to evaluate the influence of the bacterial environment on uranium extraction performance.

### 4.14 Evaluation of Dye Degradation Performance

The dye degradation performance of the *β*‐FeOOH‐coated membrane was evaluated by degrading various dyes (methyl blue, methylene blue, rhodamine B, and methyl orange) in a photochemical reaction apparatus (XPS‐7, Nanjing). After measuring the initial concentration (C_0_), the *β*‐FeOOH‐coated membrane was added to an aqueous dye solution (50 mL, 20 mg·L^−1^, pH 3). Immediately before exposure to visible light, H_2_O_2_ (9.8 mM) was introduced into the solution. Once the reaction reached equilibrium, 1 mL of the solution was extracted and diluted to 3 mL to measure the dye concentration. The dye concentrations were measured before and after the photo‐Fenton reaction to calculate the degradation efficiency. The removal ratio (R, %) was calculated using the equation:

(18) $$\mathrm{Removal}\;\mathrm{ratio}=(1\;-\frac{C_{t}}{C_{0}})\;\times \;100\%$$

where C is dye concentration after treatment of the *β*‐FeOOH‐coated membrane, and C_0_ is the initial dye concentration.

### 4.15 Oil–Water Separation Performance

The oils (petroleum ether, n‐hexane, dichloromethane, 1.3.5‐trimethyl benzene, 1.2‐dichioroethane, and Silicone oil), water (V oil: V water = 1:99), and SDS (20 mg·L^−1^) were sonicated (2 min) and magnetically stirred (12 h) to obtain homogeneous oil–water emulsions. The *β*‐FeOOH‐coated membrane was assembled in a filtration unit (10 mL), and the dead‐end filtration was conducted under a pressure of 1 bar. The flux was calculated by the following equation:

(19) $$\mathrm{Flux}=\frac{V}{A\times t\times \Delta P}$$

where F corresponds to the permeate flux (L·m^−2^·h^−1^·bar^−1^), V, A, and t refer to the permeate volume, effective membrane area, and operation time, respectively. ∆P is trans‐membrane pressure (bar).

The oil rejection ratio was calculated by the equation:

(20) $$\mathrm{Rejection}=\left(1\;-\frac{C_{t}}{C_{0}}\right)\;\times \;100\%\;$$

where Rejection denotes oil rejection, C_t_ and C_0_ refer to the oil concentrations in permeate and feed solution.

### 4.16 Self‐Cleaning Performance

After filtering the oil–water emulsion, the membrane was immersed in the H_2_O_2_ solution (35 mM) and vertically irradiated with a visible‐light source (λ > 420 nm, 500 W Xe light source) for 60 min to initiate the self‐cleaning process. Upon completion, the membrane was cleaned of residual impurities with pure water and placed into a filtration tank for a new round of oil–water separation experiments.

### 4.17 Data Analysis

The photocatalytic, adsorption, and separation experiments were performed in independent triplicates (*n* = 3). The sample size (*n* = 3) was determined a priori based on standard analytical chemistry protocols, which provides sufficient statistical power to overcome instrumental measurement variations and ensure data reproducibility. Data are presented as the mean ± standard deviation (SD). For kinetic and thermodynamic modeling, the assumption of pseudo‐first‐order, pseudo‐second‐order, Freundlich, and Langmuir was checked and validated through residual analysis. The threshold for acceptable kinetic fitting was set at a coefficient of determination (R^2^ > 0.98). Regarding the handling of missing data, a case‐wise deletion approach was employed. Any incomplete dataset caused by instrumental anomalies was discarded, and the specific experimental run was fully repeated to maintain the complete “*n* = 3” design. Statistical analyses and graphical representations were performed using IBM SPSS (version 26.0) software.

## Conflicts of Interest

The authors declare no conflicts of interest.

## Supporting information




**Supporting File**: advs76821‐sup‐0001‐SuppMat.docx.

## Data Availability

The data that support the findings of this study are available from the corresponding author upon reasonable request.
