# Supplementary material for: Metal‐Half‐Salen Chemistry for One‐Pot Mineralization on Hydrophobic Polymer Membranes
Source: Adv Sci (Weinh). 2026 Aug 2;13(59):e76821. doi: 10.1002/advs.76821 (PMC13428977; doi:10.1002/advs.76821)
Supplement: Supplementary file 1 [file ADVS-13-e76821-s001.docx]

Supporting Information

Metal-half-Salen Chemistry for One-pot Mineralization on Hydrophobic Polymer Membranes

Rou-Ming Wen^†^, Hao Ye^†^, Ming-Bang Wu*, Lu-Lin Ma, Qi-Hui Ye, Zi-Hao Yuan, Yi Xie, Juming Yao*, Hao-Cheng Yang*

Figures and Tables

**Figure S1.** ESI-MS spectra of SA-Fe^3+^ mixture.

**Figure S2.** Water contact angles of *β*-FeOOH mineralized membranes with different (a) pH, (b) Fe^3+^-SA mass ratio and (c) deposition time.

**Figure S3.** XRD pattern of modified membranes with different ligand.

**Figure S4.** TGA curves of different synthesis method.

In the TGA curve, the first mass loss stage is attributable to thermal desorption of adsorbed water and the thermal decomposition of Fe^3+^-SA complexes; the second stage is mainly caused by the conversion of *β*-FeOOH to Fe_2_O_3_^[1]^. Therefore, we can evaluate the amount of *β*-FeOOH mineralized layer on the composite membrane by the residual amount, which was calculated to be 14.94 wt% for multi-step synthesis and 16.09 wt% of ome-pot sequential synthesis.

**Figure S5.** XPS spectra of the *β*-FeOOH mineralized substrates with different etching depths.

**Figure S6.** XPS spectra of the *β*-FeOOH mineralized membranes with different deposition times.

**Figure S7.** SEM images of the *β*-FeOOH mineral layer growth.

Initial 2h image revealed particulate deposits corresponding to Fe^3+^-SA complexes anchored to the substrate through hydrophobic interactions. Subsequent deposition phases demonstrated nanoparticle nucleation mediated by Fe^3+^ ions centers within the crosslinked network, indicative of hydrolytic mineralization initiation. Prolonged deposition durations resulted in surface roughening with the in-situ growth of rod-like *β*-FeOOH microstructures.

**Figure S8.** (a) Coating thickness, (b) TGA curves, and (c) mineral content of the *β*-FeOOH mineralized substrates with different deposition time.

**Figure S9.** Coordination constants of SA ligands with different metal ions.

FeOOH mineralization step^[2]^: $\text{Fe}^{\text{3+}}\text{ + }\text{2 H}_{\text{2}}\text{O →}{\text{ }\text{FeOOH}\text{ + 3 H}}^{\text{+}}$

ZrO_2_ mineralization step^[3]^: $\text{Zr}^{\text{4+}}\text{ + }\text{8 OH}^{\text{-}}\text{→2}{\text{ }\text{ZrO}}_{\text{2}}\text{ +}\text{ 4 H}_{\text{2}}\text{O}$

MnO_2_ mineralization step^[4]^: $\text{2 Mn}^{\text{2}\text{+}}\text{ + }\text{O}_{\text{2}}\text{ +}\text{ 4 OH}^{\text{-}}\text{ → 2}{\text{ }\text{MnO}}_{\text{2}}\text{ +}\text{ 2 H}_{\text{2}}\text{O}$

**Figure S10.** ATR-FTIR spectra of modified membranes with different metals.

In ATR-FTIR spectra, various composite membranes revealed consistent characteristic peak at 1155 cm^-1^,1533cm^-1^, and 1646 cm^-1^, corresponding of N-O stretching, benzene ring skeletal vibrations, and C=N stretching attributed to SA. Corresponding metal ion coordination bonds in different metal-SA systems suggests the formation of inorganic mineral layer (673 cm^-1^: Fe-O^[5]^; 1065 cm^-1^: Zr-O^[6]^; 532 cm^-1^: Mn-O^[4]^).

**Figure S11.** (a) XPS spectra, (b) Fe 2p spectra and (c) O 1s spectra of Fe^3+^-SA system; (d) XPS spectra, (e) Zr 3d spectra and (f) O 1s spectra of Zr^4+^-SA system; (g) XPS spectra, (h) Mn 2p spectra and (i) O 1s spectra of Mn^2+^-SA system.

XPS spectra and metal element spectra of the different metal-SA systems show corresponding peaks of coordinating metal ions. For O 1s spectra, it integrates -C-O and -N-O of SA ligands, and mineral peaks from aqueous mineralization. Specially, The Mn 2p spectrum exhibited typical spin-orbit split doublets (**Figure S11h**), which were deconvoluted into Mn^3+^ (641.5 and 653.1 eV) and Mn^4+^ (644.0 and 656.5 eV) components. Notably, the distinct Mn^3+^ signal suggests a high concentration of oxygen vacancies within the mineralized MnO_2_ layer ^[4]^.

**Figure S12.** Water contact angle of modified membranes with different metals.

**Figure S13.** ATR-FTIR spectra of modified membranes with different hydrophobic membranes.

**Figure S14.** XRD patterns of modified membranes with different hydrophobic membranes.

**Figure S15.** Water contact angle of modified membranes with different substrates.

**Figure S16.** (a) Water contact angle of modified membranes and (b) UV-vis of leachate after being treated in a variety of extreme environments. (c) Ion concentration in the solution after treating the mineralized membrane in mildly acidic solution and an H_2_O_2_ solution.

**Figure S17.** (a) ATR-FTIR spectra, (b) XPS spectra and (c) U 4f spectra of Fe^3+^-SA system before and after uranium adsorption in dark.

**Figure S18.** (a) Adsorption properties of *β*-FeOOH-coated membrane with different deposition times (U(VI) concentration: 50 mg·L^-1^, volume: 50 mL, *β*-FeOOH-coated membrane mass: 10 mg). (b) Adsorption kinetics and fitted curves of physicochemical adsorption with different initial concentrations (volume: 50 mL, *β*-FeOOH-coated membrane mass: 10 mg). (c) Equilibrium isotherm adsorption of *β*-FeOOH-coated membrane fitted by Langmuir model and Freundlich model (volume: 50 mL, *β*-FeOOH-coated membrane mass: 10 mg). (d) Uranyl Selectivity of *β*-FeOOH-coated membrane (U(VI) concentration: 50 mg·L^-1^, volume: 50 mL, *β*-FeOOH-coated membrane mass: 10 mg).

A proportional increase in uranyl adsorption was observed with elevated *β*-FeOOH content in *β*-FeOOH-coated membrane Enhanced adsorption capacity and kinetics were observed with increasing uranyl concentrations, attributable to amplified concentration-driven mass transfer. Adsorption kinetics exhibited stronger correlation with the pseudo-second-order model compared to the pseudo-first-order model, indicating that uranyl extraction is governed by chemisorption mechanisms mediated through functional group coordination (**Table S4**). Adsorption isotherms under varied uranyl concentrations were modeled using Langmuir and Freundlich equations. The Langmuir model exhibited marginally higher correlation coefficients (R^2^ = 0.9828) compared to Freundlich (R^2^ = 0.9934), confirming monolayer adsorption predominance at specific coordination sites within the mineral layer (**Table S5)**. The specific coordination between uranyl ions and carboxyl functional groups enhances the material’s adsorption selectivity.

**Figure S19.** UV−vis diffuse-reflectance spectra of different membrane. (Thumbnails: Bandgap plot for the *β*-FeOOH nanorod.)

**Figure S20.** Self-made photocatalytic system.

**Figure S21.** Homogeneous reaction between U(VI) and H_2_O_2_.

It's worth noting that a control experiment involving the addition of the same amount of hydrogen peroxide, whether in light or dark conditions, demonstrated that trace amounts of H_2_O_2_ alone are ineffective in achieving substantial uranyl removal.

**Figure S22.** (a) ATP-FTIR spectra and (b) XPS spectra of *β*-FeOOH-coated membrane before and after uranium extraction. (c) U 4f spectra of *β*-FeOOH-coated membrane with different extraction times. (d) XRD pattern of *β*-FeOOH-coated membrane after uranium extraction.

The extraction of U(VI) can be divided into two processes: U(VI) (l) → U(IV) (s) → U(VI) (s). The ATR-FTIR analysis revealed a new O=U=O characteristic peak at 900.6 cm^-1^, alongside significant peak broadening at 3300 cm^-1^ (-OH) and blue-shifted Fe-O functional group (**Figure S22a**). These results confirm uranyl binding to surface functional groups and subsequent deposition ^[7]^. As shown in the XPS spectra (**Figure S22b**, **S22c**), the coating is primarily dominated by U(IV) during the pre-extraction period. As the extraction progresses, U(VI) is gradually oxidized to U(IV). The XRD results indicate that uranyl eventually binds to the membrane groups as (UO_2_)_2_O_2_∙4H_2_O in **Figure S22d** ^[8]^. Reductive extraction of uranyl dominates the fast reaction phase, whereas adsorptive accumulation governs the slow reaction phase.

Catalysis reactions of U(IV):

$\text{U(VI) +}\text{ e}^{\text{-}}\text{ → U(IV)}$

$$\text{Fe}^{\text{3+}}\text{ + }\text{e}^{\text{-}}\text{ → }\text{Fe}^{\text{2+}}$$

$$\text{Fe}^{\text{2+}}\text{ + U(VI) → }\text{Fe}^{\text{3+}}\text{ + U(IV)}$$

Reaction between U(VI) and H_2_O_2_: $\text{UO}_{\text{2}}^{\text{2+}}\text{ + 3}\text{ H}_{\text{2}}\text{O +}\text{ H}_{\text{2}}\text{O}_{\text{2}}\text{ →}\text{ (UO}_{\text{2}}{\text{)}_{\text{2}}\text{O}_{\text{2}}\text{ 4 H}}_{\text{2}}\text{O (S) +}\text{ 2 H}^{\text{+}}$

**Figure S23.** (a) Effects of different pH values on U(VI) removal ratios (U(VI) concentration: 50 mg·L^-1^, volume: 50 mL, H_2_O_2_: 9.8 mM, *β*-FeOOH-coated membrane mass: 10 mg). (b) U(VI) removal ratios of *β*-FeOOH-coated membrane at different U(VI) concentrations (volume: 50 mL, H_2_O_2_: 9.8 mM, *β*-FeOOH-coated membrane mass: 10 mg). (c) Uranium adsorption performance of *β*-FeOOH-coated membrane in uranium solution with different bacterial concentrations(U(VI) concentration: 50 mg·L^-1^, volume: 50 mL, H_2_O_2_: 9.8 mM, *β*-FeOOH-coated membrane mass: 10 mg).

**Figure S24.** (a) Recyclability of *β*-FeOOH-coated membrane (U(VI) concentration: 50 mg·L^-1^, volume: 50 mL, H_2_O_2_: 9.8 mM, *β*-FeOOH-coated membrane mass: 10 mg). (b) Uranium desorption efficiency of *β*-FeOOH-coated membrane.

**Figure S25.** EPR spectra of (a)·OH and (b) ·O_2_^-^.

The ESR test revealed that the mineralized membrane can produce a large number of hydroxyl radicals as well as superoxide radicals under light conditions. Both can generate H_2_O_2_, which interacts with the membrane to facilitate pollutant removal.

Photo-Fenton reaction^[9]^:

$$\text{Fe}^{\text{2+}}\text{ +}\text{ H}_{\text{2}}\text{O}_{\text{2}}\text{ →}\text{ Fe}^{\text{3+}}\text{ + }\text{OH}^{\text{-}}\text{ + ·OH}$$

Cycle of H_2_O_2_:

$$\text{O}_{\text{2}}\text{ + 2 }\text{e}^{\text{-}}\text{ → ·}\text{O}_{\text{2}}^{\text{-}}$$

$$\text{·}\text{O}_{\text{2}}^{\text{-}}\text{ + }\text{2 H}^{\text{+}}\text{ → }\text{H}_{\text{2}}\text{O}_{\text{2}}$$

$$\text{·OH + ·OH → }\text{H}_{\text{2}}\text{O}_{\text{2}}$$

**Figure S26.** Effect of variations in external conditions on dye degradation efficiency.

**Figure S27.** UV-vis absorption of Methyl blue (a), Methylene blue (b), Rhodamine B (c) and Methyl orange (d) before/after degradation. Insets showing changes in dyes solutions after degradation.

**Figure S28.** Bacterial growth of *Escherichia coli*, *Staphylococcus aureus* and *Bacillus subtilis* after coculture with nascent membrane, *β*-FeOOH, salicylaldoxime and *β*-FeOOH-coated membrane.

**Figure S29.** Antibacterial performances of nascent membrane, *β*-FeOOH and salicylaldoxime.

**Figure S30.** Underwater oil contact angle of *β*-FeOOH-coated membrane. The insets are oil droplet shapes on membrane surface under water.

**Figure S31.** Water flux of *β*-FeOOH-coated membrane in the pure water.

**Figure S32.** Cyclic oil (n-hexane)-water separation performance of *β*-FeOOH-coated membrane.

**Figure S33.** Water flux of *β*-FeOOH-coated membrane and nascent membrane during five pure water-bovine serum protein solution cycles.

**Table S1.** Comprehensive assessment between multi-step synthesis and one-pot sequential synthesis.

|  | PEI-PDA-*β*-FeOOH^[10]^ | TA-*β*-FeOOH^[11]^ | This work |
| --- | --- | --- | --- |
| Process | Multi-step | Multi-step | One-step |
| Waste-liquid emissions (L·m^-2^) | 21.22 | 50.00 | 2.55 |
| Chemical usage (g·m^-2^) | 149.98 | 100.00 | 56.02 |
| Reaction efficiency (h) | ≥ 24 | ≥ 24 | 2-12 |
| Environmental friendliness* | Ordinary | Ordinary | Good |

*Environmental friendliness is comprehensively evaluated based on chemical reagent consumption and wastewater discharge.

**Table S2.** Surface energy of different polymer material surfaces.

| Materials | θ_water_ (°) | θ_diiodomethane_ (°) | Surface energy |
| --- | --- | --- | --- |
| PP | 122.5 | 55.3 | 15.7 |
| PE | 104.9 | 71.1 | 18.4 |
| PTFE | 128.7 | 63.3 | 29.4 |
| PVDF | 107 | 60.9 | 26.5 |

**Table S3.** Adhesion work of the Fe^3+^-SA system on different polymer material surfaces.

| Materials | $\gamma_{s}^{d}$ (*mJ·m^-2^*) | $\gamma_{s}^{p}$ (*mJ·m^-2^*) | Adhesion work |
| --- | --- | --- | --- |
| PP | 32.0 | 2.6 | 44.9 |
| PE | 26.5 | 0.4 | 50.7 |
| PTFE | 26.4 | 3.2 | 45.2 |
| PVDF | 19.1 | 0.1 | 50.5 |

**Table S4.** The adsorption kinetic fitting parameters of the Fe^3+^-SA system with different uranium concentrations during the electro-adsorption process.

| Concentration (ppm) | Pseudo-second-order model | | Pseudo-first-order model | |
| --- | --- | --- | --- | --- |
|  | R^2^ | Q_e_ (mg g^-1^) | R^2^ | Q_e_ (mg g^-1^) |
| 10 | 0.9955 | 59.62 | 0.9947 | 49.05 |
| 25 | 0.9924 | 90.86 | 0.9883 | 80.42 |
| 50 | 0.9977 | 144.50 | 0.9919 | 130.22 |

**Table S5.** Equilibrium isotherm adsorption data fitting parameters of Fe^3+^-SA system.

| Langmuir model | | | Freundlich model | | |
| --- | --- | --- | --- | --- | --- |
| R^2^ | K_3_ (L mg^-1^) | q_m_ (mg g^-1^) | R^2^ | K_4_ (L g^-1^) | n |
| 0.9982 | 0.1 | 260.27 | 0.9828 | 5.76 | 0.662 |

# References

[1]	a) H. Yang, B. Zhu, L. Zhu, Z. Zeng, G. Wang, Z. Xiong, *ACS Appl. Mater. Interfaces* **2021**, 13, 43648-43660; b) X. T. Zhao, L. J. Cheng, N. Jia, R. X. Wang, L. F. Liu, C. J. Gao, *J. Membr. Sci.* **2020**, 600, 117857; c) H. Piao, J. Zhao, M. Liu, S. Zhang, Q. Huang, Y. Liu, C. Xiao, Chem. Eng. J. **2022**, 450, 138204.

[2]	a) L. Zhang, Y. He, L. Ma, J. Chen, Y. Fan, S. Zhang, H. shi, Z. Li, P. Luo, *ACS Appl. Mater. Interfaces* **2019**, 11, 34487-34496; b) N. Liu, R. X. Qu, Y. N. Chen, Y. Z. Cao, W. F. Zhang, X. Lin, Y. Wei, L. Feng, L. Jiang, *Nanoscale* **2016**, 8, 18558-18564.

[3]	a) H. C. Yang, Y. F. Chen, C. Ye, Y. N. Jin, H. Y. Li, Z. K. Xu, *Chem. Commun.* **2015**, 51, 12779-12782; b) H.-J. Zhou, G.-W. Yang, Y.-Y. Zhang, Z.-K. Xu, G.-P. Wu, *ACS Nano* **2018**, 12, 11471-11480.

[4]	a) X. B. Yang, Y. X. Li, D. Wu, L. L. Yan, J. Z. Guan, Y. J. Wen, Y. P. Bai, B. B. Mamba, S. B. Darling, L. Shao, *Proc. Natl. Acad. Sci. U. S. A.* **2024**, 121, e2319390121; b) X. B. Yang, Y. J. Wen, Y. X. Li, L. L. Yan, C. Y. Tang, J. Ma, S. B. Darling, L. Shao, *Adv. Mater.* **2023**, 35, 2306626.

[5]	a) Y. Chen, X. Liu, W. Wang, X. Duan, Y. Ren, *Chem. Eng. J.* **2025**, 504, 158926; b) A. T. Xie, J. Y. Cui, J. Yang, Y. Y. Chen, J. D. Dai, J. H. Lang, C. X. Li, Y. S. Yan, *J. Mater. Chem. A* **2019**, 7, 8491-8502.

[6]	a) M. Zhang, M. Xiao, W. Qi, H. Mao, *Sep. Purif. Technol.* **2024**, 332, 125665; b) J. Ran, H. Xie, X. Lai, H. Li, X. Zeng, *Tribol. Int.* **2018**, 128, 204-213.

[7]	a) X. Wu, T. Liu, H. Li, Y. He, G. Yang, W. Zhu, T. Chen, *Sci. Bull.* **2024**, 69, 3042; b) Y. Huang, M. Su, D. Chen, L. Zhu, Y. Pang, Y. Chen, *J. Hazard. Mater.* **2021**, 402, 123800.

[8] a) K. Yu, L. Tang, X. Cao, Z. Guo, Y. Zhang, N. Li, C. Dong, X. Gong, T. Chen, R. He, W. Zhu, *Adv. Funct. Mater.* **2022**, 32, 2200315; b) W. Yao, G. Zhou, T. Liu, Y. Yuan, H. Wang, N. Wang, *Adv. Sci.* **2026**, 13, e18948.

[9]	a) X. Qian, Y. Wu, M. Kan, M. Fang, D. Yue, J. Zeng, Y. Zhao, *Appl. Catal. B-Environ.* **2018**, 237, 513-520; b) S. Tian, Y. Zhang, Q. Sha, X. Zhang, T. Yang, X. Yan, N. Han, *Chem. Eng. J.* **2024**, 485, 150069; c) K. Wan, T. Fang, W. Zhang, G. Ren, X. Tang, Z. Ding, Y. Wang, P. Qi, X. Liu, *Chem. Eng. J.* **2023**, 465, 143018.

[10] Y. Lv, C. Zhang, A. He, S.-J. Yang, G.-P. Wu, S. B. Darling, Z.-K. Xu, *Adv. Funct. Mater.* **2017**, 27, 1700251.

[11] Q.-Z. Zhong, S. Li, J. Chen, K. Xie, S. Pan, J.J. Richardson, F. Caruso, *Angew. Chem. Int. Edit.* **2019**, 58, 12563-12568.
